# Introducing Care Ethics into Humanitarianism Comment on "A Crisis of Humanitarianism: Refugees at the Gates of Europe"

**DOI:** 10.15171/ijhpm.2020.14

**Published:** 2020-02-02

**Authors:** Hara Kouki

**Affiliations:** Open Lab, University of Newcastle, Newcastle upon Tyne, UK.

**Keywords:** Refugee ‘Crisis, Humanitarianism, Care Ethics, Solidarity Movement, Greece

## Abstract

Since 2015, the so-called refugee crisis has transformed ‘humanitarianism’ into a word devoid of meaning or value for European politics. By now, we all know there are numerous migrant populations in Europe living under inhuman conditions and denied their inalienable human rights; still, it seems futile to argue that equal value should be attached to all lives. Introducing care ethics into relief work calls to reflect upon humanitarianism differently, as a relationship between local communities, Non-Governmental Organization (NGO) workers and refugees that is embedded in space and time and might be allowed to have a future.

## A(nother Yet) Crisis of Humanitarianism: Refugees at the Gates of Europe

Against a backdrop of border securitization and the stigmatization of those who cross them, does it still make sense to talk about humanitarianism? Can we develop a language and a politics beyond catastrophe or victimization so as to restore our respect for all those who migrate? Can we reclaim and defend their inalienable human rights?

In her brief but concise piece, Fotaki^1^ makes a clear point that looks simple but is becoming all the more difficult to endorse: we all depend on each other so as to survive individually and as social beings; as a result, ethics of care and solidarity are genuinely political as they respond to how we share our lives, especially in times of acute environmental crisis. Humanitarianism, thus, is interrelated with politics and should be restored as a universal priority: therefore, in face of the recent so-called refugee crisis, it goes without saying that we must protect the human rights of all those who move, as their survival is at risk.

Fotaki starts by describing the events of the summer of 2015, when “more than a million refugees and forced migrants … crossed into the European Union (EU) by land and sea.” The assumption underlying official discourse since then has been that incoming populations represent a threat to the EU that should be avoided at any cost. The asylum processing became even more restrictive aiming to demarcate those “deserving” of movement and protection from those “undeserving,” who are to be returned. As a result of this exhausting process, which is required so as to become visible as a human being, the concept of the refugee is being diminished.^2^ But the changes also refer to where the control is to take place. In 2015 the EU decided to open the now infamous “hotspots” in both Italy and Greece that in practice function as camps for individuals awaiting for long periods a decision in dehumanizing conditions.^3,4^

At the same time, this has produced clear-cut hierarchies within Europe, as both countries bear an unequal burden as “gatekeepers,” which are to detain and prevent human flows from moving to the countries of the North. Especially Greece, the country hardest hit by austerity, where for both native and migrant populations terms such as “human rights” have been weakened. While thousands of people remain stranded in the country against their will, official political discourse glorifies the solidarity of EU citizens: humanitarian crisis management paradoxically combines exclusion, fear and control and experiences of suffering and signs of compassion.^5,6^

## Transforming Critique Into Action: Caring About Humanitarianism

There is an expanding scholar literature, thus, agreeing with and further developing Fotaki’s argument: the way the humanitarian system “manages” the recent incoming flows in the South of Europe produces itself blatant inequalities and rights abuses. Despite its limitations and flaws, though, there is also consensus on the fact that protecting the lives of strangers is something that should be respected and fostered. The urgent question that arises, is how to transform this critique into collective political action, how to broaden the ethics of humanitarianism making it (more) meaningful nowadays. Fotaki concludes her powerful piece by calling for “attaching equal value to all lives” that means at the same time recognizing “our dependence on others for our own survival as individuals and social beings.” This brief commentary starts from the Fotaki’s endpoint about the ethics of care, to call for reflection upon the future of humanitarianism.

Humanitarian work related with refugee “crises” is understood as a contract between governments and international, large non-governmental organizations (NGOs) and organizations that have to provide short-term relief work aiming to save peoples’ lives in emergency contexts.^7^ This has become a professionalized, standardized, often commercialized procedural task performed by humanitarian workers. Still, in practice people stay in camps much longer than they are supposed to, everyday needs and activities exceed the mandate of humanitarian organizations and host communities are profoundly affected. In real time, the logic of temporariness that structures humanitarianism is experienced by the refugees, NGO workers as a permanent condition defining their expectations, relationships and spaces. People keep moving while remaining in limbo and, as a result, a system that has been introduced to reduce suffering provokes trauma: crisis is no longer an exceptional condition. Against this “new normal” that generates powerlessness, what difference would an ethics of care make?

To begin with, an ethics of care is about an ontology of connection^8^ and emphasizes interdependence, mutuality and relationality. Humanitarian work, on the other hand, has gradually become a bureaucratic, rationalized routine in which also the biographies, affects and grievances of those who assist and spend months or years alongside migrant populations are disregarded. In Greece, living in limbo inside the camps without a clear understanding of existing restraints or possibilities challenges the (mental) health of these migrants. At the same time, EU funds have transformed NGOs into major employers for thousands of young professionals in the country otherwise facing mass unemployment, such as social workers, lawyers, doctors, and interpreters.^9^ Still, working with highly vulnerable populations under emergency circumstances and in brutal living conditions, without any prior expertise, can be extremely tough. While burnout is common among those who work with asylum seekers, in the case of precarious employees in Greece this pressure is augmented by working overtime on short-term contracts, with uncertain if any future prospects, in understaffed infrastructures in remote islands, and under unequal labor conditions compared to their colleagues from abroad.^10^ Working “in limbo” to care for traumatized people also puts at risk the (mental) health of carers and affects their work. Understanding humanitarianism not as an abstract principle or a one-way process, but as the product of relationships embedded in specific sites can reveal ways to transform policies that produce inequality.

Beginning with a mode of relationality, care ethics at the same time is flexible enough to cater for local contexts, changing needs and informal practices. In 2015, against a background of bureaucratic mechanisms and institutional standards, a massive, self-organized solidarity movement emerged in Greece.^11^ This spanned throughout the country, ranging from individuals offering food, clothes and water in remote border areas, to large squats providing shelter to hundreds of people: solidarity was about maintaining life and, for this reason, became an act of resistance against EU policies. This mobilization was rooted in the huge wave of grassroots solidarity that had developed throughout the financial crisis so as to support socioeconomically deprived people residing in the country through soup kitchens and social pharmacies, solidarity schools and markets without middlemen.^12,13^ In the social clinics, volunteer doctors, dentists, pharmacists and support staff provided free medical assistance, medicines and tests to the unemployed, uninsured and poor, including migrant populations.^14,15^ Most importantly, in these spaces patients were attended holistically and not only in relation to their symptoms; they were offered a place to talk and relate to others and were encouraged to participate in the collective running of the clinics. These initiatives offered an alternative (model of) healthcare, of enacting humanitarianism militantly.

An ethics of care is not about individualizing or exoticizing solidarity, but most often a practice that we can learn from^16^ so as to imagine the possibilities of inclusion differently. Against an urgency rationale that decontextualizes people’s lives, humanitarian work can be enriched by local practices of coexistence, solidarity and mutual support.

Providing the humanitarian system with the possibility to extend beyond the confines of urgency means at the same time introducing temporality, allowing people to have a future but also recognizing their traditions and histories. For instance, the current “refugee crisis” was the latest, but surely not the first and not the last, episode in a long history of population movements that have defined the formation of European states, including the Greek one. One could examine the refugees arriving in Greece from Asia Minor in the 1910s and 1920s as a result of population exchanges or conflict; or the “refugees” or “repatriates” from the former Soviet Union in the 1990s: in all cases “refugees” were seen – and saw themselves – as others distinct from the “Greeks.”^17^ Throughout its existence, the Greek state has been at the crossroads of population movements that have challenged its boundaries, raising issues concerning housing, health, welfare policies and labor. The challenge has also been conceptual: who “deserves” to be protected, recognized by the state and thus included in the political community. To date, research on the “refugee” phenomenon always proceeds from the assumption that this constitutes an exception to a rule that is supposedly defined by the normal state of affairs; the failure to see the “crisis” as part of a long process has resulted in failures in how state and non-state actors have responded to it.^18^ Understanding national history also as the product of the encounter between indigenous society and those seen historically as “others” can offer an alternative framing of the current “crisis” as well as another route into shaping future selves.

Care ethics is increasingly adopted lately by scholars from a range of disciplines reflecting the need to invent new methodologies and practices of coexistence and to imagine a more just world to live in. Introducing care into humanitarianism is not about adding yet another moral or ethical dimension to it, rather than suggesting an alternative politics in which we are all involved.

## Ethical issues

Not applicable.

## Competing interests

Author declares that she has no competing interests.

## Author’s contribution

HK is the single author of the paper.
